# P01.21. Effect of acupuncture on isotonic contraction of elbow flexor

**DOI:** 10.1186/1472-6882-12-S1-P21

**Published:** 2012-06-12

**Authors:** Y Kaneko, E Furuya, A Sakamoto

**Affiliations:** 1 Tokyo Medical University / Oriental Medicine Clinical Laboratory, Shinjuku, Tokyo, Japan

## Purpose

The aim of this study is to clarify the effect of acupuncture stimulation on muscular power of isotonic contraction of the elbow flexor.

## Methods

Subjects are 20 healthy males. Maximum Voluntary Contraction (MVC) of elbow flexor was measured using a strain gauge prior to exercise (MVC1). Subjects received acupuncture stimulation before the exercise on their biceps brachii. The stimulation is either deep needling (targeting muscle; mACU) or superficial needling (sACU). The exercise protocol contains 5 sets * 10 repetitions of maximum contraction of elbow flexion at 50% MVC and 90 second intervals between sets. After the exercise subjects were measured for their MVC again (MVC2). The muscle power and the velocity of every contraction was measured using a dynamometer. Electromyogram (EMG) and muscle blood flow (MBF) were also observed during the exercise. Subjects did the same exercise and we measured the same parameter without any stimulation as control. The decrease of MVC between MVC1 and MVC2, muscle power and velocity among sets and repetitions were evaluated.

## Results

MVC2 was significantly decreased compared to MVC1 in mACU and sACU and control. MVC1 of mACU seemed suppressed compared to control but no difference was observed at MVC2. Muscle power and velocity were also decreased in latter sets and repetitions in every group and the difference between mACU and sACU are under investigation.

## Conclusion

It is suggested that acupuncture stimulation may have an affect on muscle power and velocity of repeated explosive isotonic contraction. At the field or bedside, acupuncturists should treat athletes carefully before exercise or sports events.

